# From Signal Stacking to Dynamic Coupling: A Critical Review of Wearable EEG–EMG Fusion Brain–Computer Interfaces for Stroke Rehabilitation

**DOI:** 10.3390/mi17091013

**Published:** 2026-08-27

**Authors:** Mengna Dai, Mingke Jiao, Yuheng Wang

**Affiliations:** 1 Department of Medical Equipment, Chongqing Yubei People’s Hospital, Chongqing 401120, China; 2 School of Electrical and Electronic Engineering, Chongqing University of Technology, Chongqing 400054, China; 3 Department of Medical Engineering, Second Affiliated Hospital of Army Medical University, Chongqing 400038, China

**Keywords:** brain–computer interface, wearable neuroengineering, EEG–EMG integration, neuromuscular coupling, signal processing, deep learning, stroke rehabilitation

## Abstract

This structured critical review examines wearable brain–computer interface (BCI) systems that integrate electroencephalographic (EEG) and electromyographic (EMG) signals for post-stroke motor rehabilitation. The central engineering problem is the spatio-temporal heterogeneity between cortical and muscular signals, which limits the reliability and generalizability of conventional EEG–EMG fusion. We review acquisition and synchronization methods, data-, feature-, and decision-level fusion, deep-learning architectures, wearable implementation, and clinically oriented closed-loop rehabilitation. Conventional fusion can exploit complementary information but usually treats the cross-modal relationship as fixed. By contrast, dynamic brain–muscle coupling is defined here as the explicit, time-resolved estimation of interaction strength, delay, directionality, or network topology between cortical regions and target muscles. Measurable candidates include time-resolved corticomuscular coherence, phase locking, lagged dependence, information-theoretic directionality, and dynamic graph connectivity. Coupling-aware and graph-based methods are promising, but clinical translation remains constrained by artifacts, inter-subject and cross-session variability, overfitting, limited clinical datasets, interpretability, synchronization error, and embedded-computing requirements. The review therefore proposes a transparent pathway from static signal stacking toward physiologically grounded, dynamically coupled, and adaptively controlled rehabilitation systems.

## 1. Introduction

Stroke remains a major cause of long-term disability. The World Stroke Organization Global Stroke Fact Sheet 2025 reports that in 2021 there were approximately 11.9 million incident strokes and 93.8 million people living with stroke worldwide, while stroke caused about seven million deaths globally [1]. Upper-limb impairment commonly restricts activities of daily living, while rehabilitation technologies and treatment delivery remain heterogeneous across clinical settings [2,3].

Brain–computer interface technology offers a promising adjunct to post-stroke rehabilitation by translating neural or neuromuscular activity into feedback, robotic assistance, or functional-electrical stimulation [4,5,6,7,8,9,10]. EEG captures cortical dynamics related to movement preparation, imagery, and attempted movement, whereas EMG reflects residual or executed muscular activation. Their integration can therefore monitor the intention–execution pathway more comprehensively than either modality alone [4,5,6,7].

Many existing systems nevertheless follow a signal-stacking paradigm: EEG and EMG are processed separately and then combined through feature concatenation, weighted voting, or other static fusion rules [11,12]. Such architectures can improve classification, but they do not necessarily model the evolving physiological relationship between brain and muscle. Physiological transmission delay, independent device clocks, spatially diffuse EEG, localized EMG, and post-stroke reorganization can all change the measured cross-modal relationship across time, participants, sessions, and tasks [5,13,14,15,16,17]. Representative clinical/core and contextual/enabling evidence is summarized in Table 1 after the review methodology.

Unlike broad reviews of BCI neurorehabilitation [8,18], hybrid EEG-based multimodal interfaces [11], or functional-electrical-stimulation control systems [19], this review uses the operational distinction between static signal fusion and time-resolved brain–muscle coupling as its organizing framework. A fundamental reorientation is therefore required—from asking only how signals are fused to asking what physiological interaction is being estimated. This review makes four contributions: (i) it reports a transparent search, eligibility, and citation-audit procedure; (ii) it defines dynamic brain–muscle coupling in physiological, computational, and measurable terms; (iii) it compares synchronization and decoding strategies using structured tables; and (iv) it identifies the engineering and clinical evidence required for closed-loop translation. The argument is not that conventional fusion is ineffective, but that static fusion alone is insufficient when the clinically relevant target is the evolving brain–muscle interaction itself.

The remainder of the review proceeds from acquisition and synchronization to algorithmic fusion and coupling, wearable implementation, clinical evidence, limitations, and future directions. Numerical results are reported only when they can be traced to the cited primary source, and adjacent non-stroke studies are explicitly identified as enabling rather than clinical evidence.

### Review Methodology and Citation Audit

This article is a structured critical review rather than a prospectively registered systematic review. Searches were conducted in PubMed/MEDLINE, Web of Science Core Collection, Scopus, and IEEE Xplore; Google Scholar and backward/forward citation tracking were used to identify additional records. The principal time window was January 2016 to 31 July 2026, with earlier foundational methodological papers retained only when required to define graph attention or physics-informed learning. Database-specific syntax was adapted around four core concept blocks: (stroke OR post-stroke) AND (EEG OR electroencephalography) AND (EMG OR electromyography) AND (hybrid BCI OR multimodal BCI OR corticomuscular coupling OR brain–muscle coupling). Supplementary terms covered wearable systems, synchronization, deep learning, graph neural networks, and closed-loop rehabilitation.

Records were screened first by title and abstract and then by the available full text or a sufficiently informative abstract. Publications were eligible when they addressed concurrent or complementary EEG–EMG acquisition; motor-intention decoding or brain–muscle coupling; wearable, robotic, haptic, virtual-reality, or functional-electrical-stimulation rehabilitation; or an enabling electrode, synchronization, artifact-control, adaptation, or embedded-computing method with direct relevance to wearable neurorehabilitation. Duplicate records, publications unrelated to neuromotor or wearable biosignal systems, and records without sufficient methodological information were excluded. Non-stroke studies were retained only as explicitly labeled enabling evidence.

For each included publication, evidence was extracted into predefined fields covering population, sample size, stroke stage, acquisition modality, synchronization strategy, feature or coupling metric, learning algorithm, fusion level, validation protocol, reported performance, clinical outcome, follow-up, and main limitation. Direct stroke and EEG–EMG evidence was synthesized separately from contextual or enabling evidence. Because the tasks, populations, comparators, and outcomes were heterogeneous, no pooled meta-analysis or cross-study performance ranking was attempted.

**Figure 1:**
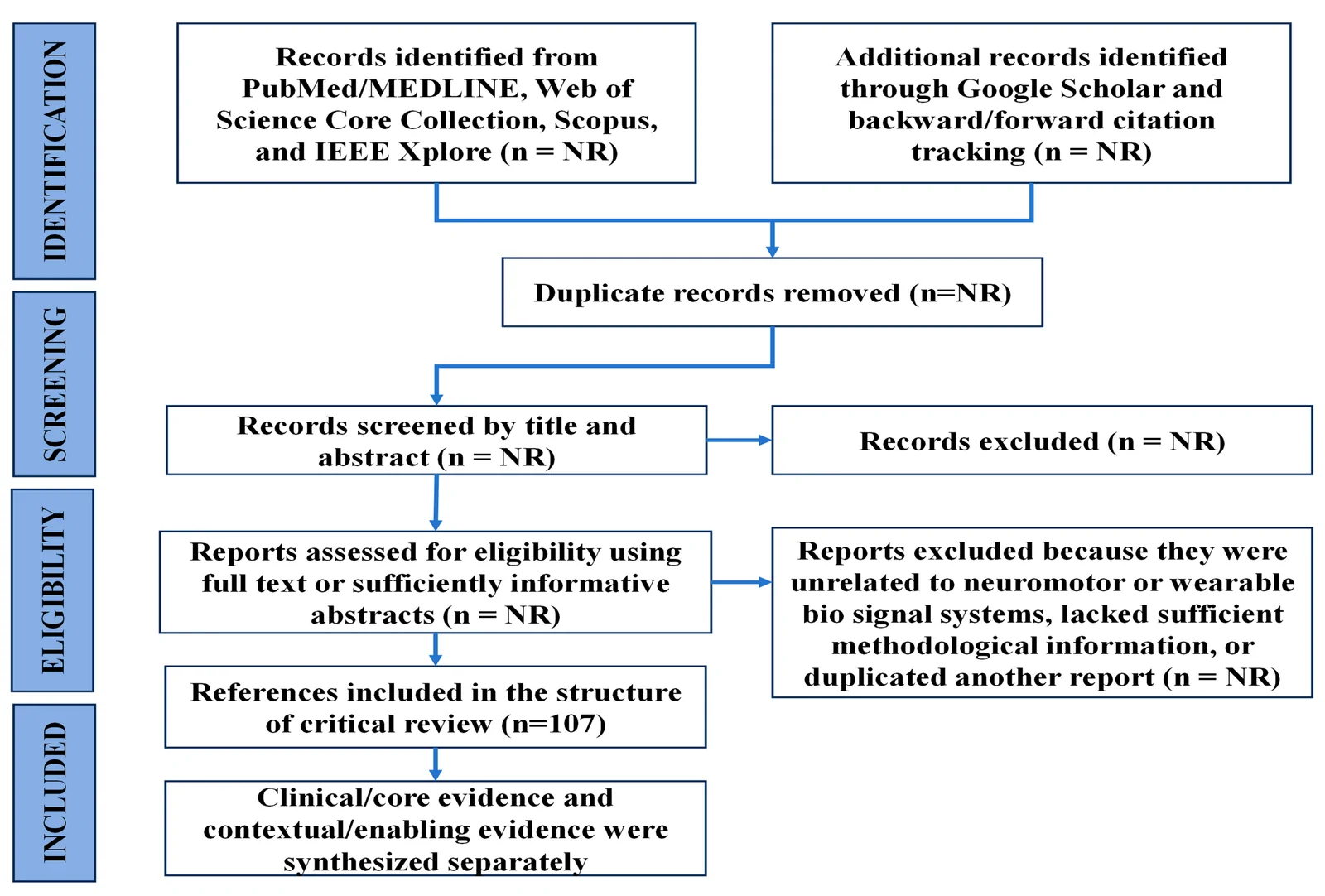
Literature identification and selection process for the structured critical review. NR indicates that the corresponding count was not retrospectively recoverable because the original record-level database exports and deduplication logs were not prospectively archived. The principal search window was January 2016 to 31 July 2026, with earlier foundational methodological papers retained when necessary.

The final corpus comprised 107 publications. Searches were documented at the database and keyword levels, but the original record-level exports and deduplication logs were not prospectively archived. Initial retrieval, duplicate screening, and stage-specific exclusion counts therefore cannot be reconstructed reliably and are reported as not retrospectively recoverable (NR), rather than inferred. Each in-text citation was audited against the publication title, venue, year, abstract, or full text where available, and the specific claim it supports. Figure 1 summarizes the documented selection process and explicitly displays the unavailable counts. The diagram follows PRISMA 2020 reporting principles for transparency, but this structured critical review is not presented as prospectively registered or fully PRISMA-compliant [20].

**Table 1:** Representative clinical and enabling evidence for wearable EEG–EMG BCIs, including sample characteristics, validation, outcomes, and follow-up.

Ref.	Year	Population, Sample, and Stroke Stage	Signals/Intervention	Study Design and Validation	Reported Outcome and Follow-Up	Main Limitation
[21]	2016	Conference abstract; sample size and stroke stage not reported.	EEG–EMG corticomuscular-coupling feature.	Proof-of-concept; covariate-shift adaptation; validation incompletely reported.	Personalized brain–muscle adaptation; no quantitative clinical outcome or longitudinal follow-up.	Abstract-level evidence; insufficient detail for reproducible quantitative comparison.
[22]	2018	30 severely affected patients; chronic stroke.	Surface EMG vs. ipsilesional sensorimotor-rhythm EEG detector.	Within-dataset comparison using an existing randomized-trial dataset.	Residual EMG detectable in 22/30 (73%); no separate longitudinal follow-up.	Modality comparison; no explicit brain–muscle coupling.
[5]	2023	12 patients (subacute-to-chronic) + 12 healthy participants.	Concurrent 61-channel EEG + EMG from eight muscles per arm.	Offline high-density CMC network comparison; associations with FMA and MMT.	Affected-hand network weight/density differed and correlated with impairment; no longitudinal follow-up.	Small sample; offline analysis; no external validation.
[7]	2023	Public dataset: five healthy volunteers + two patients with stroke; 516 and 174 trials, respectively.	Synchronized 32-channel EEG + 8-channel EMG during push–pull tasks.	GIN; 4:1 train/test split; cross-subject evaluation.	88.89% accuracy vs. 73.23% benchmark; no therapeutic or longitudinal follow-up.	Only two patients with stroke *; no prospective clinical validation.
[23]	2023	Public WAL–EEG–GAL dataset; clinical status, sample size, and stroke stage incompletely reported in abstract.	EEG–EMG hybrid hand-rehabilitation decoding.	GCN–LSTM for EEG; CNN for EMG; source-dataset evaluation.	Mean EEG/EMG accuracies 0.892/0.954; no clinical follow-up.	No external, cross-session, or prospective clinical validation.
[17]	2022	13 healthy participants + 12 patients with stroke *.	Concurrent EEG–EMG CMC during grasping and extension.	Pseudo-online sliding-window CMC; 125-ms updates.	Stroke cohort: ~85% hit rate; ~580-ms mean delay; no longitudinal follow-up.	Pseudo-online; sensitive to timing and window selection.
[9]	2020	Fifty-one patients: forty-five chronic, six subacute.	Motor-imagery EEG–BCI + FES + virtual-reality feedback.	25 sessions/3 months; pre, post, 1- and 6-month assessments.	Mean FMA–UE +4.68 points; gains maintained at 1 and 6 months.	No concurrent randomized control group.
[24]	2021	Ten severely impaired patients: seven subacute, three chronic.	EEG motor-imagery BCI + robotic hand orthosis.	Randomized crossover: 12 BCI sessions + 12 conventional-therapy sessions.	Both periods improved FMA–UE/ARAT; no between-treatment difference; no post-crossover follow-up.	Small sample; no washout; possible carryover.
[25]	2025	52 early-subacute ischemic stroke patients randomized; 48 completed (25 BCI, 23 control).	Eight-channel EEG + visual-feedback/pedaling robot; EMG and fNIRS assessment.	Double-blind RCT; 10 real-time vs. sham-feedback sessions over 2 weeks.	FMA–UE change 4.0 vs. 2.0 points (*p* = 0.046); immediate post only.	Short intervention; no later follow-up or external replication.
[4]	2025	35 patients with severe chronic upper-limb paralysis after stroke.	EEG, residual EMG, and combined EEG–EMG features for attempted-movement decoding.	Offline secondary analysis; rest vs. movement-attempt classifiers.	Combined EEG–EMG improved decoding and showed complementary information; no therapeutic follow-up.	No online intervention or longitudinal recovery assessment.
[6]	2025	Conference report; sample size and stroke stage not reported in accessible abstract.	CMC-informed hybrid BCI + FES.	CMC patterns before/after a 1-month intervention.	Reduced maladaptive patterns reported; no follow-up beyond intervention.	Conference-level reporting; limited sample, statistical, and replication detail.
[18,26,27,28,29,30,31]	2016–2025	Grouped reviews, invasive studies, physiological cohorts, and datasets; heterogeneous populations/sample sizes.	EEG, EMG, ECoG, fMRI, and related enabling modalities.	Contextual/enabling evidence; no common validation protocol.	Rehabilitation physiology/dataset-design context; heterogeneous follow-up.	Not all wearable EEG–EMG clinical trials; direct ranking inappropriate.

* For Ref. [17], *n* = 11 for the extension-movement analysis and *n* = 12 for grasping. Abbreviations: ARAT, Action Research Arm Test; BCI, brain–computer interface; CMC, corticomuscular coherence; CNN, convolutional neural network; ECoG, electrocorticography; EEG, electroencephalography; EMG, electromyography; FES, functional electrical stimulation; FMA, Fugl–Meyer Assessment; FMA–UE, Fugl–Meyer Assessment of the Upper Extremity; fMRI, functional magnetic resonance imaging; fNIRS, functional near-infrared spectroscopy; GCN–LSTM, graph convolutional network–long short-term memory; GIN, graph isomorphism network; MMT, Manual Muscle Test; RCT, randomized controlled trial; VR, virtual reality. “No longitudinal follow-up” indicates that outcomes were not assessed after the study-specific intervention or recording period.

## 2. Engineering Foundations of EEG–EMG Integration

### 2.1. EEG Signals: Physiological Basis, Acquisition Technologies, and Feature Extraction

Electroencephalography provides millisecond-scale access to cortical dynamics relevant to movement preparation and execution. Motor-imagery studies commonly analyze event-related desynchronization/synchronization in the μ and β bands [32,33], whereas movement-related and transition-related temporal structure provides complementary information about impending movement [13,34]. Connectivity and amplitude features can also be combined for EEG-driven arm-movement decoding [35]. These features support motor-intention decoding but are sensitive to task design, preprocessing, and participant-specific neurophysiology.

Wet EEG electrodes generally provide stable low-impedance contact but require conductive media and preparation time; dry or limited-channel systems improve portability at the cost of greater sensitivity to motion and contact variability [33,36]. MXene-enabled hydrogel interfaces and other conformal materials can improve electrode–skin coupling [37], while the open-source BEATS platform illustrates high-precision multichannel acquisition [38]. EEG quality can be degraded by cap motion, physiological artifacts, ballistocardiographic contamination, volume conduction, and source-localization uncertainty [39,40,41,42,43]. Artifact-removal methods must therefore be evaluated on realistic dynamic recordings rather than assumed to be neutral preprocessing [40,41,42,43,44].

For wearable motor decoding, feature selection should match the physiological question. ERD/ERS and other movement-related temporal features characterize cortical state changes [13,32,33,34], whereas deep models can learn task-specific temporal representations. However, high classification accuracy in a laboratory EEG dataset does not by itself demonstrate reliable brain–muscle coupling or clinical utility; participant-independent and session-independent evaluation remains necessary [31,36,45].

### 2.2. EMG Signals: Physiological Basis and Acquisition Technologies

EMG reflects the summed electrical activity of recruited motor units and provides direct information about muscle activation, residual movement intent, recruitment, and fatigue. HD-sEMG and decomposition methods can characterize motor-unit discharge behavior non-invasively [46,47], while the physiological limitations of surface EMG—including volume conduction, crosstalk, electrode placement, and amplitude cancellation—must be considered when interpreting coupling [48].

Modern sEMG systems integrate low-noise amplification, filtering, analog-to-digital conversion, and wireless transmission [49,50]. Flexible and soft electrodes improve conformity and long-term stability [49,51,52]. Hybrid EMG–FMG systems add mechanical information about muscle force and deformation [53,54], but these signals should not be treated as interchangeable with EMG because their physiological origins and delays differ.

Time-domain descriptors such as RMS amplitude, zero crossings, and waveform length, and frequency-domain descriptors such as median or mean power frequency, are widely used to quantify activation and fatigue [48,50,55,56,57]. Gesture-decoding studies further demonstrate how feature design, classifier choice, and acquisition conditions affect performance [58,59,60]. Muscle fatigue, forearm angle, electrode shift, and acquisition time introduce non-stationarity [56,61], and domain adaptation may be required when hardware or sessions change [62].

In rehabilitation, EMG can quantify residual muscle function, trigger assistance, or provide a peripheral target for closed-loop control [22,23,63,64]. The critical distinction is whether EMG is used as an independent classifier input, a label or training aid, or an explicit physiological partner in a coupling estimate. These uses have different synchronization, interpretation, and validation requirements [5,7,16,17].

### 2.3. Synchronization Challenges in Concurrent EEG–EMG Acquisition

Simultaneous EEG and EMG acquisition is essential for estimating corticomuscular interaction, but observed delay combines physiological conduction, acquisition latency, buffering, clock drift, resampling, and algorithmic windowing [13,14,15,16,17,65]. Motion artifacts, power-line interference, and shared mechanical contamination can create spurious cross-modal dependence [41,44,66]. Consequently, synchronization error is not merely a data-management issue: it can bias phase, coherence, lag, directionality, and graph-edge estimates.

Four synchronization strategies are commonly used. Shared-clock or hardware-trigger designs provide the strongest temporal reference but require integrated hardware [14,65]. Trigger pulses can estimate fixed offsets between independent systems [65]. Timestamp correction compensates for packet-level delay and clock drift, while application-layer alignment methods such as simple data alignment and linear-interpolated data alignment reconstruct a common sampling grid in BLE systems [15]. Software-only alignment is flexible but cannot fully recover timing information lost through unsynchronized sampling or packet loss. Signal-quality and synchronization diagnostics should therefore be stored with the biosignals [14,15,65,67,68].

The most appropriate synchronization architecture depends on the target metric. Classification based on long windows may tolerate modest residual error, whereas phase-sensitive corticomuscular coherence, short-latency movement detection, and directed coupling require tighter timing control [5,16,17]. Ultra-conformal electrodes and integrated acquisition frameworks can improve signal stability [67,68], but they do not replace an explicit common time base. Table 2 compares the timing fidelity, latency implications, limitations, and coupling suitability of the principal synchronization strategies.

Direct numerical ranking of synchronization methods is not yet defensible because the cited studies report different combinations of offset, drift, jitter, packet delay, alignment-window length, and processing latency. Future wearable EEG–EMG studies should report these components separately, together with inference and actuator delay, rather than presenting a single unspecified latency value [14,15,17,65].

## 3. From Fusion to Coupling: Algorithmic Paradigm Evolution

### 3.1. Multimodal Signal Fusion: Theoretical Framework and Inherent Limitations

Conventional multimodal integration can occur at the data, feature, or decision level [11,12]. Data-level fusion combines synchronized samples or representations before modality-specific abstraction; feature-level fusion concatenates or transforms extracted features; and decision-level fusion combines independently generated outputs. These categories describe where information is combined, but not whether the physiological relationship between modalities is explicitly estimated.

EEG and EMG are complementary because cortical activity can precede or persist without overt movement, whereas EMG provides peripheral evidence of attempted or executed contraction [4,7,13,22]. Static fusion can improve robustness when one modality is noisy, and adjacent applications such as EEG–facial-EMG pain analysis, physiological emotion recognition, and EMG gesture recognition illustrate the broader value of multimodal features [60,69,70]. However, performance gains in non-stroke tasks should not be interpreted as direct evidence of rehabilitation efficacy.

Attention mechanisms, recurrent networks, adversarial alignment, and tensor representations can learn complex cross-modal features [71,72,73,74]. Nevertheless, adaptive weighting is not automatically equivalent to dynamic physiological coupling. A model may change feature weights over time while still lacking an interpretable estimate of interaction strength, delay, directionality, or network topology.

Physiologically, dynamic brain–muscle coupling denotes time-varying coordination between cortical population activity and muscle recruitment across movement preparation, initiation, execution, and sensorimotor feedback. It may reflect descending drive, common input, or feedback-related reorganization, but correlation alone does not establish causality. Computationally, for cortical signal x_i_ and muscle signal y_j_, a local coupling state can be written as C_ij_(t,f) = M({x_i_(τ), y_j_(τ): τ ∈ W_t_}), where M is a time-localized association estimator and W_t_ is a sliding or event-locked window. Depending on the hypothesis, M may quantify corticomuscular coherence, phase locking, lagged dependence, directed information transfer, or a dynamic graph edge [5,13,16,17].

A system qualifies as dynamically coupled only when the interaction state is estimated repeatedly and enters the model as an explicit input, latent state, output, or control variable. Operational indicators include coupling magnitude and peak frequency, phase or delay, directionality, edge topology, and stability across windows. Reports should specify window length, update interval, sampling and synchronization error, artifact controls, amplitude dependence, test–retest reliability, and added value beyond static fusion. Graph-based sequential decoding is an enabling architecture [7], but learned attention or graph weights should not be interpreted as physiological coupling without independent validation. Table 3 summarizes these operational distinctions.

**Figure 2:**
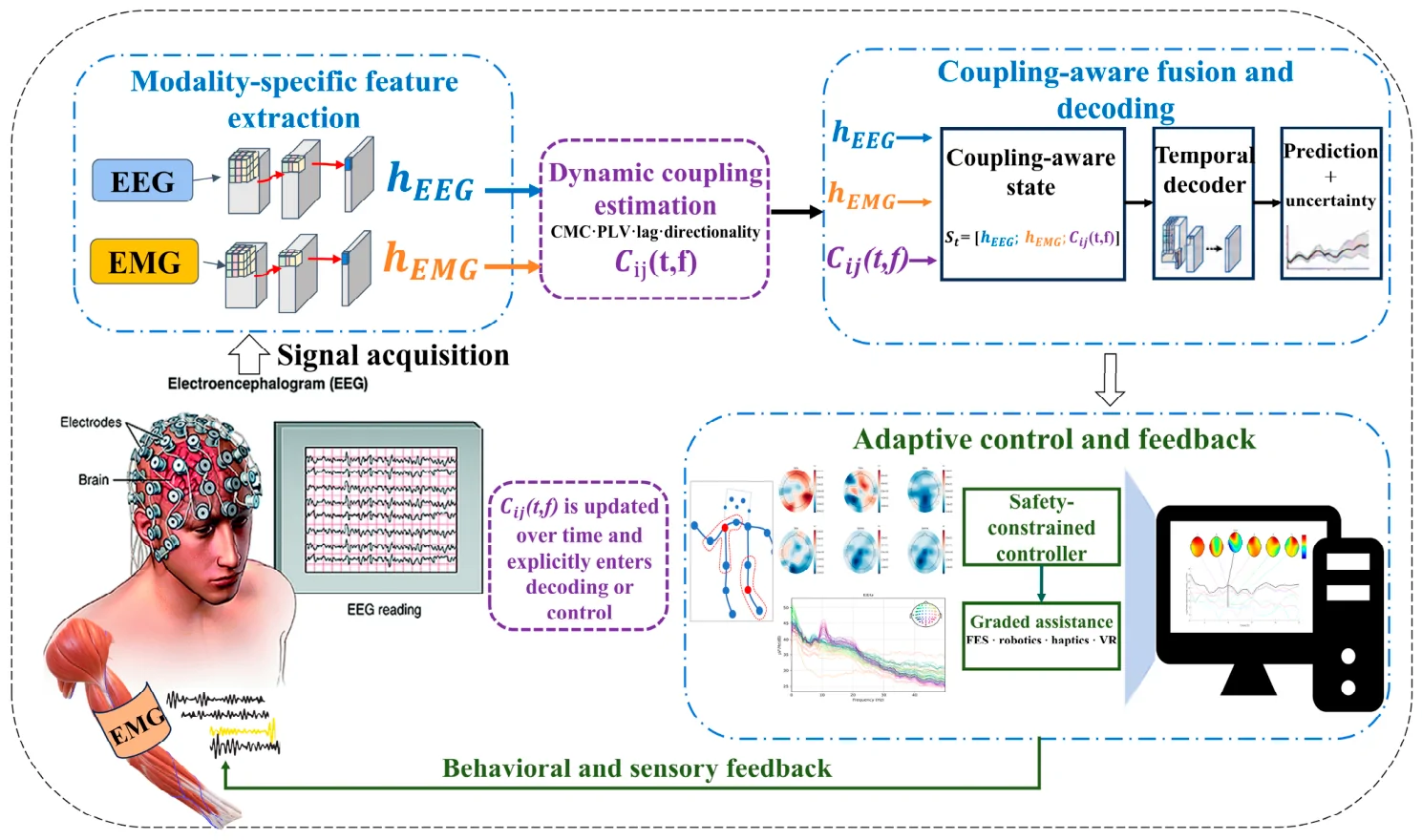
Proposed dynamic-coupling framework for wearable EEG–EMG BCI rehabilitation. Synchronized EEG and EMG signals are encoded separately, while a time-varying coupling state Cij(t,f) is estimated using corticomuscular coherence, phase locking, lagged dependence, directionality, or dynamic graph edges. The coupling state is explicitly integrated with modality-specific features for temporal decoding, uncertainty estimation, and safety-constrained adaptive control. Behavioral and sensory feedback closes the rehabilitation loop.

Figure 2 presents the proposed dynamic-coupling architecture. Synchronized EEG and EMG are encoded separately, while a time-varying interaction state Cij(t,f) is repeatedly estimated and combined with modality-specific representations for temporal decoding, uncertainty estimation, and safety-constrained adaptive control. Unlike a conventional static-fusion pipeline, the coupling state enters both decoding and closed-loop control, and behavioral and sensory feedback completes the rehabilitation loop.

### 3.2. Traditional Machine Learning: Limitations of Static Feature Representations

Traditional machine-learning methods remain important baselines because they are comparatively transparent and computationally efficient. SVMs, random forests, and related classifiers can operate on engineered EEG, EMG, or multimodal features [60,70,75]. Their performance is highly dependent on preprocessing, feature definition, participant selection, and validation protocol; results from emotion or gesture datasets should not be generalized to stroke rehabilitation without external clinical validation.

The main limitation of handcrafted pipelines is not simply lower capacity, but the assumption that a fixed vector adequately represents a non-stationary neuromuscular process. Vectorization can discard temporal and graph structure [73], while fatigue, posture, session drift, and participant heterogeneity alter the feature distribution [61,62,75]. These models remain valuable as interpretable comparators and for low-power deployment, but coupling claims require explicit interaction features and timing validation.

### 3.3. Deep Learning: A Bridge to Dynamic Coupling

**Figure 3:**
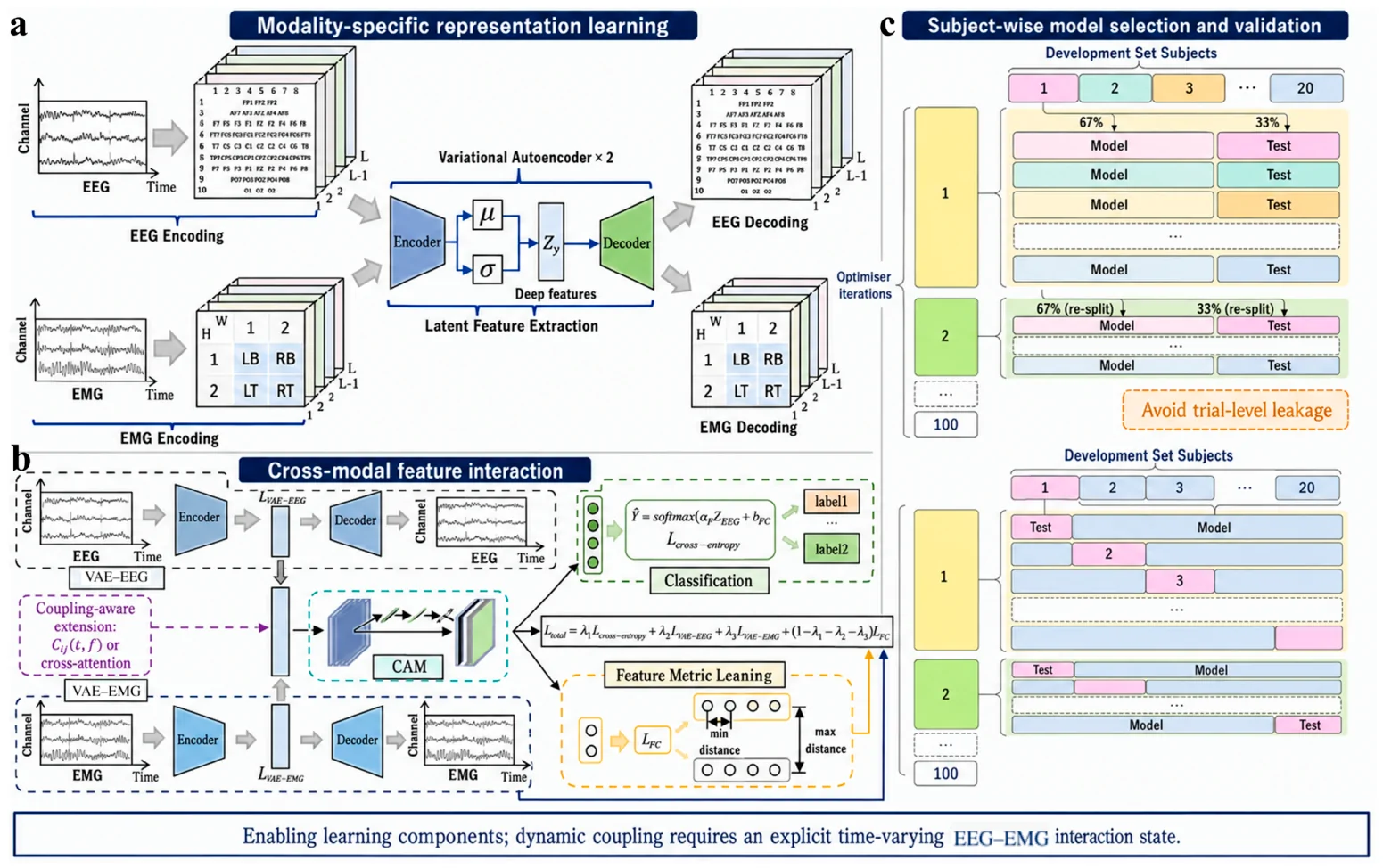
Representative deep-learning components that may support coupling-aware EEG–EMG analysis. (**a**) Modality-specific representation learning using the 2M-hBCINet architecture [74]; (**b**) VAE-based EEG–EMG deep-feature extraction and cross-modal feature interaction [74]; and (**c**) subject-wise data flow for bespoke and generalist fusion-model selection [12]. These architectures represent enabling learning and validation components; explicit dynamic coupling additionally requires a time-varying EEG–EMG interaction state, such as Cij(t,f). In panel (**c**), ellipses denote omitted intermediate subject indices and repeated validation iterations. Arrows indicate the direction of data flow, while colors distinguish different modalities, learning modules, and validation subsets and do not represent quantitative values.

**Figure 4:**
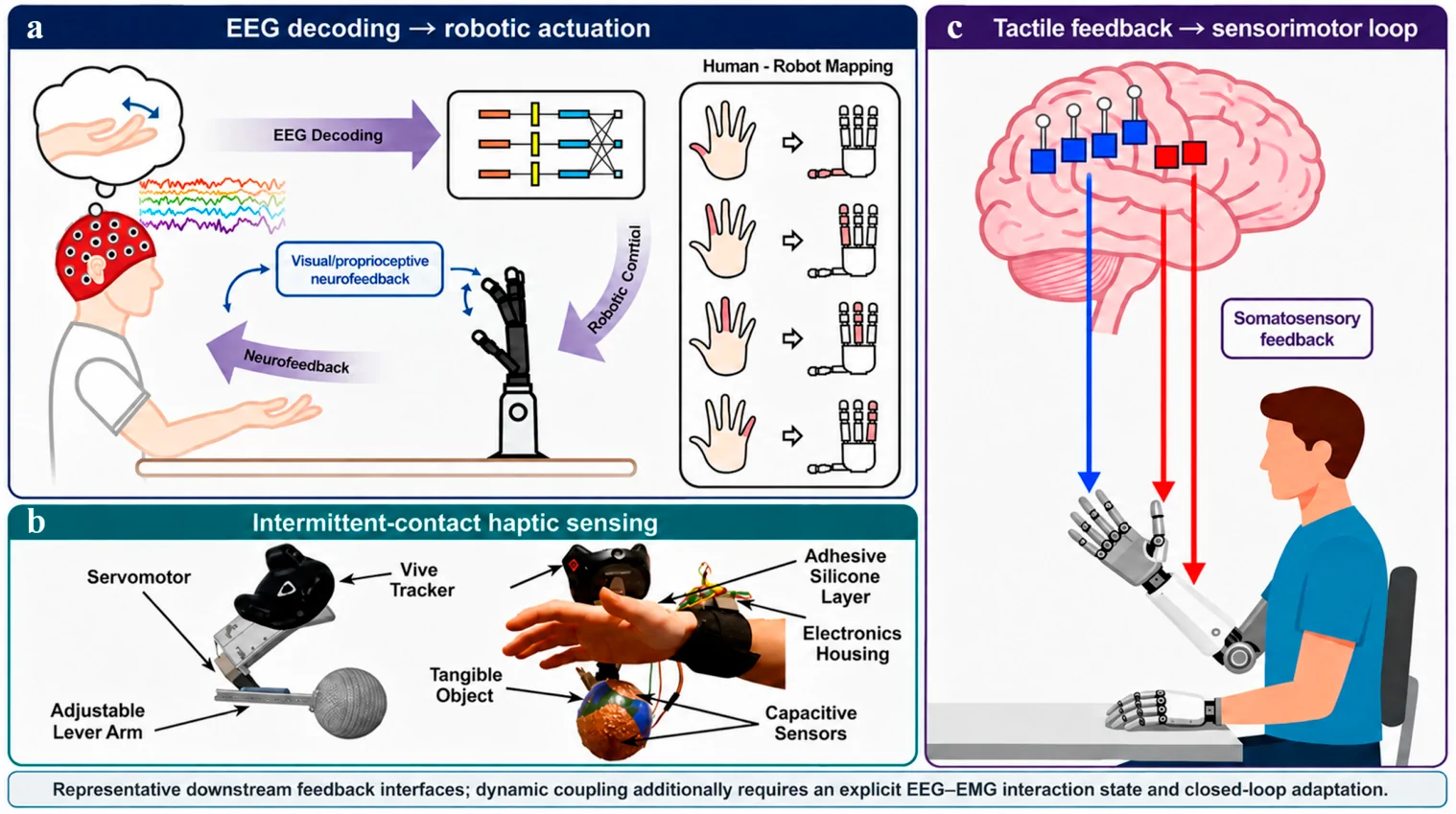
Representative downstream closed-loop and haptic-feedback interfaces. (**a**) EEG decoding for robotic-hand actuation and visual/proprioceptive neurofeedback [80]; (**b**) intermittent-contact haptic sensing [81], with cropping and rearrangement for layout; and (**c**) tactile feedback supporting a sensorimotor loop adapted from [80,82]. These examples illustrate downstream interfaces and should not be interpreted as evidence of dynamic EEG–EMG coupling unless an explicit interaction state is estimated and incorporated into control. Arrows indicate the direction of control or sensory-feedback flow; colors are used only to distinguish schematic pathways and do not encode quantitative values.

Deep learning can learn hierarchical spatial and temporal representations and thereby provide building blocks for coupling-aware models. DenoiseMamba combines convolutional and state-space components for EEG artifact removal [76]. CNN-LSTM approaches can decode muscle-related information from spatiotemporal EEG [77], while DSCNN and Flashlight-Net illustrate efficient convolutional designs for SSVEP and motor-imagery EEG [78,79]. These studies are enabling examples rather than paired post-stroke EEG–EMG coupling studies. Figure 3 summarizes representative multimodal learning mechanisms, whereas Figure 4 illustrates downstream robotic and haptic feedback contexts; neither architecture should be treated as evidence of dynamic coupling unless an explicit interaction state is estimated.

Channel and cross-modal attention can emphasize task-relevant EEG and EMG representations. In 2M-hBCINet, variational autoencoders, channel attention, and multitask objectives are combined to learn deep EEG–EMG features [74]. Attention weights can aid model inspection, but they should not be interpreted as physiological connectivity without independent validation against coupling metrics.

Temporal models, including recurrent networks and adversarial-alignment architectures, can capture sequence structure and reduce modality mismatch [72,83]. Their validation should distinguish within-subject, cross-subject, cross-session, and external-dataset performance because random trial-level splits can substantially overestimate clinical generalizability.

End-to-end, semi-supervised, multitask, and transfer-learning approaches can reduce dependence on manual feature engineering and calibration [74,75,84,85]. Real-time EEG decoding frameworks such as NeuroGrasp and smartphone EEG cleaning demonstrate implementation feasibility [86,87], but neither is an EEG–EMG dynamic-coupling model because an explicit interaction state is not estimated.

### 3.4. Efficiency, Robustness, and Generalization in Dynamically Coupled Systems

Dynamic models increase computational and memory demand because coupling must be recomputed across channels, frequency bands, and sliding windows. Heterogeneous ARM-FPGA architectures demonstrate that quantization, pipelining, and hardware acceleration can reduce BCI latency [88], while wearable-system reviews describe trade-offs among power, heat, bandwidth, and model complexity [11,89,90]. Reports should therefore separate acquisition, preprocessing, coupling estimation, inference, communication, and actuator latency and should provide memory, power, and thermal measurements under the deployed configuration.

Robustness requires standardized terminology, reproducible preprocessing, and explicit artifact control. Electrooculography (EOG) should be used to characterize ocular contamination, while motion, ocular activity, and EMG leakage into EEG can produce shared variance that resembles coupling [41,44,76,87]. Filter-bank common spatial pattern (FBCSP) is a feature-extraction method rather than evidence of cross-modal coupling. Multimodal fusion may improve classification [12], but coupling should be computed only after artifact sensitivity, synchronization, and preprocessing choices have been evaluated.

Generalization across participants and sessions requires adaptation, recalibration, uncertainty reporting, or transfer learning [21,62,75,85]. An adjacent EEG-biometric study used EMG-driven additive augmentation with triplet-loss training [29], illustrating a possible data-augmentation strategy rather than evidence of post-stroke therapeutic efficacy. Architectures originating in adjacent EEG domains, including residual-gated multimodal Transformers [91], multiband convolutional networks [92], and decomposition pipelines [93], likewise illustrate transferable engineering options. Invasive ECoG remapping in individuals with upper-limb paralysis [26] is physiologically informative but should not be treated as evidence for wearable scalp EEG–EMG systems. Explainable-AI approaches may help relate learned features to neurophysiology [94]. Table 4 compares the modality, feature strategy, fusion or coupling level, validation, reported performance, computational complexity, and clinical limitations of representative method classes. Because tasks and cohorts differ, performance is reported without imposing a cross-study rank order.

## 4. Wearable Systems and Closed-Loop Rehabilitation Applications

### 4.1. Hardware Platforms for Dynamic EEG–EMG Coupling

Wearable hardware is the physical foundation of dynamic EEG–EMG coupling because the algorithm can only estimate interaction from synchronized and stable signal pairs. Design priorities include low-noise acquisition, common timing, miniaturization, low power, thermal safety, and mechanically stable electrode–skin interfaces [89,90]. Flexible arrays and conformal electrodes improve contact and reduce motion-related impedance changes [67,96,97], while wearable displays may support interactive feedback [98].

EEG and EMG differ in amplitude, bandwidth, channel density, and artifact susceptibility. A shared platform must therefore provide modality-appropriate gain and filtering without saturating either front end, while preserving a common time base. Miniaturized hardware improves portability [90], but long-duration use also requires stable placement, low heat, skin compatibility, and reproducible contact [67,96,97].

Wireless transmission introduces trade-offs among throughput, latency, packet loss, and power. BLE is practical for wearable networks, but clock drift and buffering require timestamp correction or explicit alignment [14,15,65]. LPWAN and embedded machine learning can extend range and edge intelligence [99], while multistream platforms illustrate the need to store synchronization and data-quality metadata [100]. A wearable decoder should report packet loss, drift, and end-to-end latency rather than only classification accuracy.

Modularity and standardized interfaces facilitate maintenance and sensor replacement, but modularity must not fragment the timing architecture. Reproducible software frameworks can improve multimodal deployment [68], and integrated physiological acquisition systems provide practical design templates [63,100,101]. Biocompatibility, cleaning, electrode reuse, and electrical safety should be evaluated alongside algorithmic performance [67,97].

### 4.2. Rehabilitation Paradigms: From Passive Triggering to Active Modulation

In this review, EEG–EMG integration denotes the coordinated use of cortical and muscular signals, whereas a hybrid EEG–EMG BCI denotes a system in which both modalities contribute to decoding, assessment, or control. EEG can provide evidence of movement preparation or attempt, while EMG indicates residual or executed muscle activation [4,22,23,64]. Simultaneous classification can improve intention–execution assessment, but coupling-aware control additionally requires an explicit time-varying interaction estimate [5,7,17].

**Figure 5:**
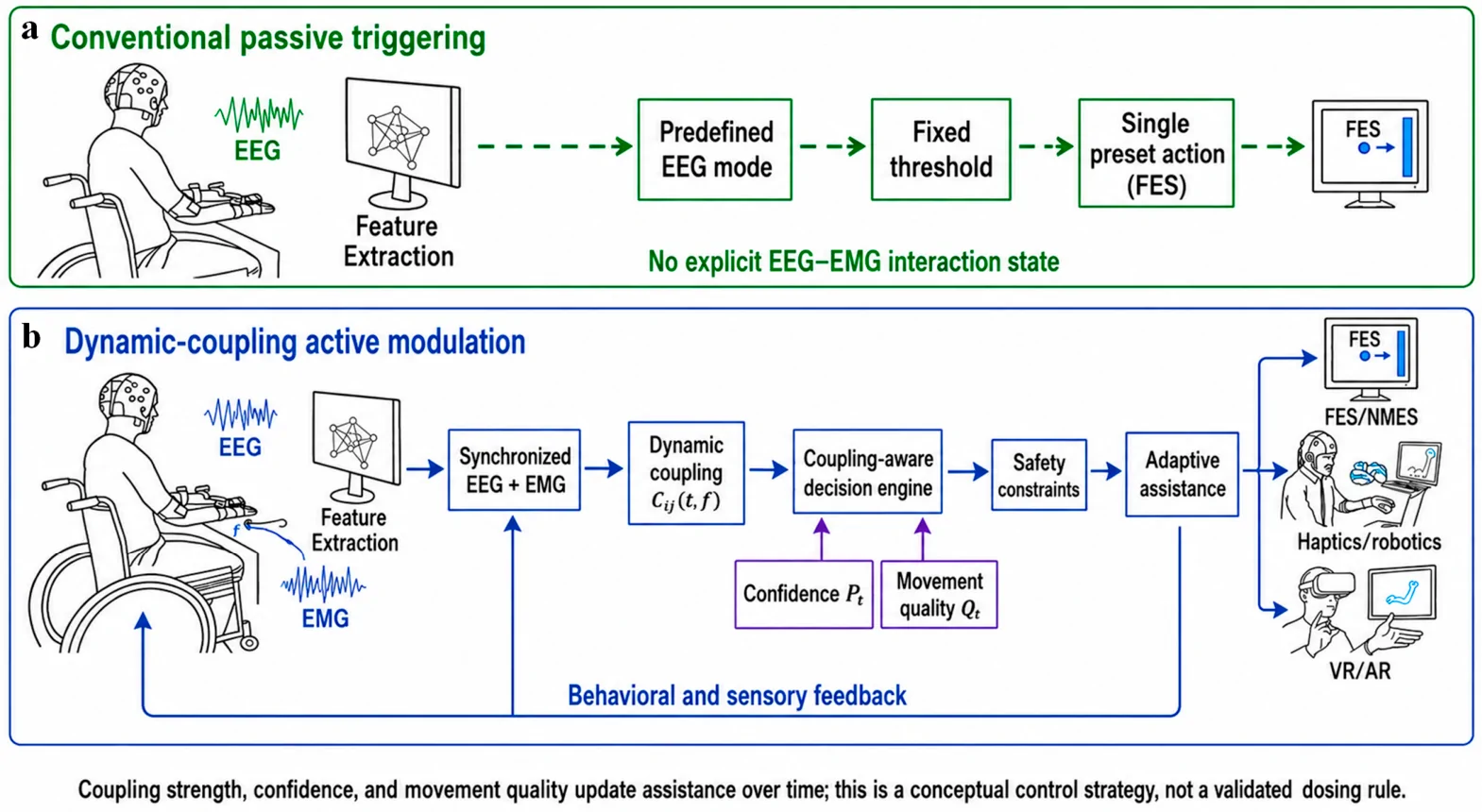
Conceptual comparison between conventional passive triggering and dynamic-coupling-based active modulation. (**a**) In the conventional pipeline, features extracted from EEG are used to identify a predefined EEG mode, which is evaluated against a fixed threshold to trigger a single preset action, illustrated here by functional electrical stimulation (FES); no explicit time-varying EEG–EMG interaction state is estimated. (**b**) In the proposed active-modulation framework, synchronized EEG and EMG signals are used to estimate a dynamic coupling state, C_ij_(t,f). The coupling state, prediction confidence P_t_, and movement-quality indicator Q_t_ jointly inform a coupling-aware decision engine, safety constraints, and adaptive assistance delivered through FES/neuromuscular electrical stimulation (NMES), haptic or robotic interfaces, or virtual/augmented reality (VR/AR). Behavioral and sensory feedback close the control loop adapted from [6,7,17,19]. This author-developed framework represents a conceptual control strategy rather than a clinically validated control or dosing algorithm. Dashed green arrows indicate the conventional fixed-trigger pathway; solid blue arrows indicate dynamic signal, control, and feedback flow; and purple arrows indicate confidence and movement-quality inputs. The colors are schematic and do not represent quantitative values.

Robotic orthoses, exosuits, wheelchairs, virtual-reality environments, haptic interfaces, and functional-electrical stimulation can translate decoded activity into graded assistance or feedback [19,24,80,82,102,103,104]. In passive triggering, a thresholded EEG or EMG event initiates a fixed action. In active modulation, assistance is updated according to coupling strength, delay, confidence, movement quality, or task performance. Evidence from these device classes is heterogeneous, so system feasibility should be distinguished from durable clinical recovery. Figure 5 contrasts fixed triggering with the proposed coupling-aware active-modulation framework.

In the proposed paradigm, coupling strength, confidence, and movement quality jointly update adaptive assistance and multimodal feedback. Because this is an author-developed conceptual strategy rather than a validated dosing rule, future trials must define safe thresholds, adaptation rates, failure handling, clinician override criteria, and whether stronger coupling should increase or decrease assistance for a given task [6,7,8,17,19].

### 4.3. User Experience and Device Usability

User experience is an engineering outcome because preparation time, comfort, appearance, perceived control, and reliability determine whether a wearable BCI is used repeatedly. User-centered work emphasizes simplified setup, acceptable aesthetics, therapist compatibility, and transparent feedback [2]. Signal instability can reduce trust; lightweight denoising and few-channel wearable systems may improve usability but require realistic online validation [87,95,105].

Adherence should be evaluated through donning time, cognitive load, discomfort, skin tolerance, session completion, social acceptability, and the perceived relationship between the device and therapist [2,24]. These outcomes should be reported together with algorithmic metrics because a highly accurate laboratory model can still fail clinically if it is difficult to wear, calibrate, or understand.

Human–machine interfaces should minimize operating steps and present feedback that is interpretable to both patients and therapists. Robotic orthosis, exosuit, wheelchair, and haptic studies provide practical examples of visual, proprioceptive, and tactile feedback [24,80,82,103,104]. Interface design should also include confidence or signal-quality warnings so that assistance is not changed on the basis of corrupted data.

### 4.4. Clinical Evidence and Outcome Evaluation

Clinical evidence remains heterogeneous. A systematic review of 25 upper-limb FES studies reported favorable Fugl–Meyer outcomes for manually controlled, BCI-controlled, and EMG-controlled FES, with the largest pooled mean difference reported for EMG-controlled FES (14.14; 95% CI, 11.72–16.60); an ARAT mean difference of 11.9 (95% CI, 8.8–14.9) was also reported for EMG-controlled FES [19]. These results support the broader closed-loop rehabilitation concept, but they do not prove that EEG–EMG dynamic coupling is superior to conventional control.

Across intervention studies, the evidence supports feasibility and short-term motor benefit but remains heterogeneous in design and strength (Table 1). The Sebastián-Romagosa feasibility study provides longitudinal evidence that BCI-associated gains can persist beyond training, whereas the Cantillo–Negrete crossover study did not demonstrate superiority over conventional therapy. The more recent randomized trial by He et al. provides stronger controlled evidence of short-term benefit, but its brief intervention and lack of post-treatment follow-up limit conclusions about durability [9,24,25]. Collectively, these studies support BCI-assisted rehabilitation as a plausible therapeutic platform, but they do not establish that dynamic EEG–EMG coupling is the mechanism responsible for clinical improvement.

Coupling-specific evidence is less mature and is dominated by offline or pseudo-online analyses rather than prospective therapeutic trials (Table 1). High-density CMC network studies link brain–muscle coordination with impairment, pseudo-online CMC demonstrates the feasibility of coupling-informed control, and graph-based EEG–EMG decoding shows that joint representations can improve task classification [5,7,17]. However, small stroke cohorts, limited cross-session or external validation, and absent longitudinal intervention outcomes prevent these findings from establishing dynamic coupling as a validated therapeutic target. The larger attempted-movement analysis by López-Larraz et al. strengthens the evidence that EEG and residual EMG carry complementary information, but it remains an offline decoding study rather than a coupling-controlled intervention [4]. Exoskeleton-related connectivity findings are therefore treated as contextual rather than direct coupling evidence [27].

Multimodal clinical assessment can combine behavioral scales with EEG, EMG, fNIRS, or imaging markers to characterize functional change and neuroplasticity [5,25,30]. Clinical trials should prespecify primary outcomes, adverse events, adherence, and follow-up, and should distinguish decoder performance from durable motor recovery. External validation, longitudinal monitoring, and standardized reporting are required before coupling-derived measures can be considered clinical biomarkers.

## 5. Challenges and Limitations

The principal barriers can be grouped into signal reliability, model generalization, embedded implementation, and clinical evidence. These issues are interdependent: synchronization and artifacts bias coupling; small datasets increase overfitting; complex models increase latency and power demand; and poor usability limits the repeated longitudinal data needed for personalization. The following three subsections therefore distinguish signal-integrity problems, algorithmic and system-level bottlenecks, and clinical-translation barriers while making their interactions explicit.

### 5.1. Signal Integrity and Data Reliability

Motion artifacts, ocular activity, muscle contamination of EEG, weak post-stroke EMG, electrode displacement, and power-line interference can all generate false cross-modal dependence [41,44,66,76]. Studies should report excluded segments, channel rejection, synchronization failures, signal-quality thresholds, and sensitivity to preprocessing. Coupling metrics should be recomputed under plausible preprocessing alternatives to demonstrate that conclusions are not artifact-driven.

### 5.2. Algorithmic and System-Level Bottlenecks

Algorithmic bottlenecks include inter-subject variability, cross-session drift, class imbalance, overfitting, interpretability, and insufficient external validation. The graph isomorphism network (GIN), a graph neural network variant, improved overall push–pull classification from 73.23% with CSP–SVM to 88.89% on the synchronized dataset [7], but that result should not be generalized directly to clinical recovery. CMC-based control is physiologically meaningful [16,17], yet it remains sensitive to window length, frequency selection, muscle activation level, timing error, and the number of usable channels.

Overfitting risk is amplified when high-dimensional windows from the same participant are randomly divided across training and test sets. Participant-grouped splits, nested hyperparameter tuning, calibration assessment, learning curves, ablation studies, and external datasets should therefore be reported where feasible [7,31,62,74]. Interpretability analyses should be stress-tested against preprocessing changes and physiologically implausible perturbations; attention maps alone are insufficient. Real-time reports should additionally disclose parameter count, memory footprint, operations per inference, device-level power, thermal behavior, and end-to-end latency [87,88,95].

### 5.3. Barriers to Clinical Translation

Clinical translation is limited by the gap between laboratory demonstrations and deployable therapeutic workflows. Model outputs must be interpretable, failure states must be visible, and assistance must remain under safe clinical control. Small single-center datasets, inconsistent stroke-stage reporting, and variable endpoints make cross-study performance ranking unreliable [2,8,25,105].

Stroke populations differ in lesion location, severity, chronicity, cognition, fatigue, residual muscle activity, and abnormal agonist–antagonist coactivation [4,5,17,22,28,45]. These factors change both signal quality and the underlying brain–muscle relationship. Stratified reporting, leave-subject-out validation, cross-session testing, uncertainty estimation, and subgroup-specific failure analysis are therefore required; random trial-level splits alone are insufficient.

Device cost and operational complexity remain major barriers. Studies should quantify setup time, usable recording duration, battery endurance, packet loss, calibration burden, skin reactions, cleaning, and therapist workload [89,90,95,105]. These system-level outcomes determine scalability and should not be replaced by isolated classification accuracy.

## 6. Future Directions Toward Dynamic Coupling BCIs

### 6.1. Explainable and Physiologically Constrained Models

Future models should combine dynamic graphs with explicit neurophysiological constraints. Graph attention provides a general mechanism for learning time-varying contributions of neighboring nodes [106], while the EEG–EMG GIN study demonstrates the feasibility of graph-structured sequential decoding [7]. Physics-informed neural networks provide a broader framework for embedding known constraints into model optimization [107]. In EEG–EMG applications, candidate constraints include plausible temporal ordering, anatomically reasonable connectivity, and consistency between descending drive and muscle activation. These are research hypotheses, not validated clinical rules.

### 6.2. Coupling Metrics as Quantitative Biomarkers

Coupling indicators should be treated as candidates rather than established biomarkers. Corticomuscular and intermuscular coupling measures have demonstrated physiological and control relevance [5,16,17], but clinical qualification requires test–retest reliability, sensitivity to recovery, independence from artifacts and signal amplitude, and prospective association with meaningful functional outcomes. Longitudinal studies should compare coupling-derived measures with Fugl–Meyer scores, task performance, and clinician-rated recovery.

### 6.3. System Integration and Large-Scale Clinical Translation

Translation will require co-design of algorithms, hardware, and trials. Hardware acceleration and miniaturized architectures can reduce latency and power [88,89,90], while flexible electrodes improve long-term acquisition [67,96,97]. Multicenter studies should use standardized synchronization metadata, external model validation, prespecified safety rules, and clinically meaningful endpoints [17,19,24,25]. The goal is not merely a smaller classifier, but a reliable closed-loop system whose assistance changes are traceable to validated physiological and behavioral indicators.

## 7. Conclusions

Wearable EEG–EMG BCIs provide complementary access to cortical movement intention and peripheral execution, but the value of the two modalities depends on how their relationship is represented. Conventional data-, feature-, and decision-level fusion can improve classification, yet it generally leaves the time-varying brain–muscle interaction implicit.

The central contribution of this review is a rigorous definition of dynamic brain–muscle coupling as a time-resolved estimate of interaction strength, delay, directionality, or topology. This definition separates explicit coupling models from adaptive weighting or deep feature concatenation and establishes measurable criteria for evaluating future systems.

Progress requires three coordinated advances: reliable synchronized wearable acquisition; coupling-aware and physiologically constrained models with participant- and session-independent validation; and clinical studies that connect coupling-derived measures to functional recovery, usability, and safety. Graph-based learning, corticomuscular metrics, transfer learning, and embedded acceleration are promising components, but none substitutes for rigorous external and longitudinal validation.

Reframing EEG–EMG integration from signal stacking toward dynamic coupling therefore provides an engineering pathway rather than a claim of completed clinical translation. The immediate research priority is to test whether time-varying coupling measures improve adaptive control and predict recovery beyond conventional features, confidence estimates, and behavioral scales.

## Figures and Tables

**Table 2:** Timing fidelity, latency implications, and limitations of synchronization strategies for concurrent EEG–EMG acquisition.

Strategy	Operating Principle	Timing Fidelity and Latency	Main Limitation	Implication for Coupling
Shared clock/integrated acquisition	A common oscillator controls simultaneous ADC timing.	Relative fidelity is highest because offset is bounded mainly by ADC timing and front-end group delay; end-to-end latency remains pipeline-dependent.	The approach increases hardware complexity and constrains channel-range design.	It is preferred for phase/coherence and short-latency coupling [14].
Hardware trigger/timing pulse	A common event is injected into both acquisition systems.	Initial offset calibration can be precise, but drift persists between triggers and event delivery adds latency.	Both devices must expose trigger access, and repeated calibration may be required.	It is useful when independent commercial devices must be synchronized [65].
Timestamp and drift correction	Local timestamps are mapped to a common clock.	Fidelity depends on timestamp resolution and clock stability; buffering and clock mapping introduce variable latency.	Performance depends on packet metadata, clock diagnostics, and drift-model validity.	It is suitable for wearable networks when offset, drift, and jitter are explicitly reported [15].
SDA/LIDA software alignment	Packet streams are aligned and, when required, interpolated onto a common grid.	At least one packet or alignment window is required; missing samples cannot be recovered, and interpolation may alter phase.	Phase and spectral estimates can be biased by packet loss and resampling choices.	It can support BLE monitoring, but coupling estimates require sensitivity analysis [15].
Post hoc physiological alignment	Signals are aligned using detected physiological events.	Timing is task-dependent and non-causal; latency is at least the event-detection window.	Correction may remove or confound the physiological delay that the analysis aims to estimate.	It is exploratory and is not preferred for causal or directed coupling inference [13,17].

**Table 3:** Operational distinction between conventional EEG–EMG fusion and dynamic brain–muscle coupling.

Dimension	Conventional Fusion	Dynamic Coupling
Primary representation	Raw samples or modality-specific features are combined.	An explicit time-varying cortical-muscular interaction C_ij(t,f) is estimated.
Temporal assumption	The cross-modal relationship is often fixed within a trial or model.	Strength, delay, directionality, or topology is allowed to evolve.
Minimum operational criterion	No explicit cross-modal interaction variable is required.	The interaction state must be repeatedly estimated and updated.
Measurable indicators	Task accuracy, fused probability, or feature importance is usually reported.	Magnitude, frequency, phase, delay, directionality, topology, and temporal stability can be reported.
Physiological interpretability	Interpretation is usually indirect.	Interpretation can be physiological only when the coupling metric and artifact controls are independently validated.
Typical outputs	Outputs include class labels, probabilities, or fused feature vectors.	Outputs include coupling trajectories, graphs, delays, directionality, and task predictions.
Principal strength	The approach is simple, computationally efficient, and provides a robust baseline.	The approach can represent adaptive neuromuscular coordination and support mechanism-aware control.
Main limitation	Interaction dynamics may be missed.	Estimates are sensitive to synchronization, artifacts, windowing, model complexity, and small datasets.
Validation priority	Task performance and robustness across participants and sessions are primary.	Timing sensitivity, negative artifact controls, test–retest reliability, physiological plausibility, and incremental value beyond static fusion are required.
Clinical role	Typical uses are movement-intention detection and fixed triggering.	Potential uses include adaptive assistance, mechanistic assessment, and candidate biomarkers.

**Table 4:** Structured comparison of representative learning and implementation strategies, including validation and reported performance.

Method/Representative Algorithm	Modality and Feature Strategy	Fusion or Coupling Level	Validation and Reported Performance	Computational Complexity	Clinical Applicability/Main Limitation
Handcrafted ML [60,70,75]	The studies use engineered EEG, EMG, or adjacent multimodal features.	Feature- or decision-level fusion is used; coupling remains implicit.	Validation is mainly within-dataset; metrics are not directly comparable across emotion, gesture, and motor-imagery tasks.	Low	The methods provide transparent baselines but lack paired stroke EEG–EMG external validation.
CNN/CNN-LSTM [77,78,79]	Raw or time-frequency EEG representations are learned; the examples are predominantly EEG-only.	A learned representation is produced without an explicit brain–muscle interaction state.	Validation is dataset-specific; high source-dataset performance does not provide a common external stroke benchmark.	Moderate–high	Representation learning is strong, but physiological coupling and cross-session robustness remain unverified.
Multitask EEG–EMG network [74]	Deep embeddings are learned from paired EEG and EMG.	VAE, channel attention, and multitask fusion are combined.	LOOCV and ablation support source-dataset feasibility, but no independent stroke cohort was evaluated.	High	The model performs explicit multimodal learning, but attention weights are not validated physiological coupling.
GIN sequential model [7]	Synchronized EEG–EMG trials are represented as sequential graphs.	Graph-level sequential fusion is learned.	A 4:1 train/test evaluation and cross-subject analysis reported 88.89% accuracy versus 73.23% for the benchmark.	High	Only two patients with stroke were included, and no prospective clinical validation was performed.
CMC pseudo-online control [17]	Concurrent EEG and EMG are converted to a sliding-window coherence feature.	CMC is used as an explicit coupling feature.	Thirteen healthy participants and twelve patients with stroke were assessed; the stroke cohort achieved about 85% hit rate with about 580 ms mean delay.	Moderate	Physiological relevance is direct, but timing sensitivity and lack of therapeutic follow-up limit translation.
Attention/adversarial/Transformer models [71,72,91]	The examples use adjacent EEG–fNIRS, workload-physiology, or sleep-neurophysiology embeddings rather than paired stroke EEG–EMG.	Attention or domain alignment produces adaptive cross-modal weighting.	Task-specific metrics are heterogeneous and cannot be compared directly with stroke EEG–EMG studies.	High	The architectures are transferable, but attention weights should not be interpreted as validated connectivity.
Adaptive/transfer learning [62,75,85]	Source and target EEG or EMG representations are aligned or fine-tuned.	Domain adaptation occurs at the feature or model level.	Cross-domain or target fine-tuning produced study-specific gains; no common paired EEG–EMG stroke benchmark was used.	Moderate	Recalibration burden may decrease, but negative transfer and domain mismatch remain risks.
Embedded/few-channel systems [87,88,95]	Single-channel cleaning, accelerated hardware, or reduced-channel EEG inputs are used.	The contribution is at the system-implementation level rather than explicit coupling.	Hardware or online metrics are device-specific; end-to-end EEG–EMG latency was not reported on a common basis.	Low–moderate	Wearability can improve, but spatial information and paired-modality evidence may be reduced.

## Data Availability

No new data were created or analyzed in this study. Data sharing is not applicable to this article.
